# Optimizing Left Atrial Appendage Occlusion: Limitations of TEE and the Emerging Role of Multimodality Imaging

**DOI:** 10.1111/anec.70113

**Published:** 2025-09-18

**Authors:** Muhammet Cihat Çelik, Mehmet Murat Şahİn, Macit Kalçık

**Affiliations:** ^1^ Department of Cardiology Hitit University Erol Olçok Education and Research Hospital Corum Turkey; ^2^ Department of Cardiology, Facult of Medicine Hitit University Corum Turkey

**Keywords:** atrial fibrillation, intracardiac echocardiography, left atrial appendage occlusion, stroke prevention, transesophageal echocardiography

## Abstract

This letter critically appraises the recent study by Long et al. investigating the role of transesophageal echocardiography (TEE) in guiding and evaluating left atrial appendage occlusion (LAAO) among patients with non‐organic heart disease. While the authors demonstrate the procedural utility of TEE, its limitations, including invasiveness, patient tolerance, and lack of long‐term data, remain notable. Current literature highlights the growing role of intracardiac echocardiography (ICE) and computed tomography (CT) as alternative or complementary modalities. Larger multicenter studies integrating these approaches are warranted to optimize procedural outcomes and patient safety in atrial fibrillation–related stroke prevention.

To the Editor,

1

We read with great interest the recent article by Long et al. that explored the application of transesophageal echocardiography (TEE) in guiding and evaluating left atrial appendage occlusion (LAAO) in patients with non‐organic heart disease (Long et al. 2025). The authors emphasized that TEE provides accurate measurements, improves procedural safety, and assists in postoperative evaluation. This contribution is valuable, especially since LAAO has become an important alternative for stroke prevention in patients who cannot use long‐term anticoagulation.

Although the study provides important insights, it is important to consider the limitations of relying solely on TEE. TEE is a semi‐invasive technique that requires sedation and may not be well tolerated by elderly or fragile patients. In recent years, intracardiac echocardiography (ICE) has been increasingly used as an alternative, offering comparable accuracy for device deployment with fewer complications and without the need for general anesthesia (Frans et al. 2025). Therefore, the exclusive focus on TEE may not fully reflect the current advances in clinical practice.

Another issue relates to the small sample size and short follow‐up in this study. Only 48 patients were included, and the postoperative evaluation period was limited to 1 month. Such a design makes it difficult to assess important long‐term outcomes, such as thromboembolic events or device‐related complications. Previous studies have shown that multimodality imaging, particularly the use of cardiac CT and three‐dimensional TEE, may improve device sizing and detection of peri‐device leaks (Hahn et al. 2022; Kandi et al. 2022). Without these comparisons, the strength of the authors' conclusions is limited.

The clinical perspective should also take into account patient comfort and resource use. TEE requires fasting, sedation, and specialized personnel, which can increase procedure costs and patient discomfort. In contrast, ICE and pre‐procedural CT imaging are less invasive and may reduce these concerns while still providing accurate anatomical information (Qu et al. 2022). In modern practice, balancing diagnostic precision with patient‐centered care is essential.

In conclusion, Long et al. highlight the important role of TEE in LAAO patients, but broader studies with larger populations and longer follow‐up are necessary. Future investigations should compare TEE with ICE and CT‐based strategies to determine the most effective, safe, and patient‐friendly imaging approach. Such evidence will guide clinicians in optimizing imaging selection for atrial fibrillation patients undergoing LAAO.

## Author Contributions

The author takes full responsibility for this article.

## Conflicts of Interest

The authors declare no conflicts of interest.

## Data Availability

The authors have nothing to report.
